# Feline and Canine Rabies in New York State, USA

**DOI:** 10.3390/v13030450

**Published:** 2021-03-10

**Authors:** Scott Brunt, Heather Solomon, Kathleen Brown, April Davis

**Affiliations:** Wadsworth Center, New York State Department of Health, Slingerlands, NY 12159, USA; scott.brunt@health.ny.gov (S.B.); heather.solomon@health.ny.gov (H.S.); labmother19@gmail.com (K.B.)

**Keywords:** rabies, vaccination, feline, canine, raccoon variant, epidemiology, New York, USA

## Abstract

In New York State, domestic animals are no longer considered rabies vector species, but given their ubiquity with humans, rabies cases in dogs and cats often result in multiple individuals requiring post-exposure prophylaxis. For over a decade, the New York State rabies laboratory has variant-typed these domestic animals to aid in epidemiological investigations, determine exposures, and generate demographic data. We produced a data set that outlined vaccination status, ownership, and rabies results. Our data demonstrate that a large percentage of felines submitted for rabies testing were not vaccinated or did not have a current rabies vaccination, while canines were largely vaccinated. Despite massive vaccination campaigns, free clinics, and education, these companion animals still occasionally contract rabies. Barring translocation events, we note that rabies-positive cats and dogs in New York State have exclusively contracted a raccoon variant. While the United States has made tremendous strides in reducing its rabies burden, we hope these data will encourage responsible pet ownership including rabies vaccinations to reduce unnecessary animal mortality, long quarantines, and post-exposure prophylaxis in humans.

## 1. Introduction

Rabies is a fatal disease that affects the central nervous system with no effective treatment once clinical symptoms begin. Worldwide, the cost of canine rabies specifically is significant; the economic expenditure is estimated at 8.6 billion dollars and accounts for 98% of the nearly 60,000 annual rabies deaths worldwide [1,2]. Over the past 50 years, the United States has put forth significant efforts toward the eradication of canine rabies in animal and human populations. The creation of animal vaccination protocols and the implementation of leash laws have reduced the number of animals infected with canine rabies from up to 10,000 cases per year to the last known case in 2004 [3]. Despite this advancement, rabies remains persistent in wildlife reservoirs (among raccoons, skunks, and foxes) in the United States and continues to be a serious public health risk. The possibility of cross-species transmission to domestic animal populations increases the opportunity for disease emergence in humans due to the multidimensional linkage domestic animals present in modern society. From 2004 to 2018, an average of 72 dogs and 279 cats were diagnosed with rabies in the United States annually [4,5,6,7,8,9,10,11,12,13,14,15,16,17,18], nearly all after contact with a wild animal. Human rabies cases, which once fluctuated between 30 to 50 infections yearly, have since decreased to approximately 3 deaths per year, and nearly all were acquired either domestically from bats or overseas from dogs [4,5,6,7,8,9,10,11,12,13,14,15,16,17,18].

According to the American Veterinary Medical Association, 38.4% of households own dogs, making them the most common companion animal with nearly 77 million reported in the United States [19]. Cats are a close second, with 25.4% of households reporting ownership [19]. Although over 58 million cats live in households, it is estimated that there are anywhere from 30 million–100 million feral cats throughout the United States [20]. Trap-neuter-vaccinate-return (TNVR) programs were intended to reduce feral populations; however, free-roaming cat populations make it difficult to differentiate an owned outdoor pet from one of a truly stray or feral status. Although companion animals are required to be vaccinated for rabies in New York State, many owned cats are not vaccinated despite the risk of being exposed to rabid wildlife outdoors or the possibility of a rabid animal entering the home, such as a bat. Since the control of canine rabies in the United States, cats have become the most common domestic animal to contract rabies and the fifth most common species after bats, raccoons, skunks, and foxes [18]. Since 1988, the number of rabid cats diagnosed with rabies in the US annually has surpassed the number of rabid dogs [21].

Between 2004 and 2018, cats testing positive for rabies in the US ranged from 241 to 319 cases annually. A review found that 44% and 32% of individuals in Pennsylvania and New York, who received post-exposure prophylaxis (PEP) did so following an exposure to a cat [22]. For example, when a stray kitten was diagnosed with rabies in New Hampshire in 1994, 665 people received PEP [23]. Although this case is extreme, exposures to rabid or potentially rabid dogs and cats often result in multiple individuals requiring PEP [22,24,25]. Administration of PEP in large-scale situations may present an additional challenge as rabies PEP is expensive, at times in short supply, and not without potential adverse effects although it is generally well tolerated. In New York State, PEP consists of human rabies immune globulin (HRIG) administered at 20 IU/kg body weight and four doses of rabies vaccine on days 0, 3, 7, and 14. For those with a history of rabies vaccination, only the vaccine is administered on days 0 and 3.

In the study described here, the New York State Rabies Laboratory investigated the rabies emergence risks in vaccinated and unvaccinated companion animals, their ownership status, and their likely route of exposure via variant typing.

## 2. Materials and Methods

### 2.1. Data and Demographics

The New York State Department of Health (NYSDOH) Wadsworth Center Rabies Laboratory receives approximately 6500 animals for diagnostic testing annually. This primarily includes a wide variety of animals including raccoons, skunks, foxes, cats, dogs, livestock, and several species of bats. Our overall positivity rate on all specimens is about 6.5%. Prior to specimen submission, laboratory protocol requires that complainants complete a history form on all animals undergoing diagnostic testing. Although some information fields are often not filled out properly, that is not immediate grounds for specimen dismissal. As delays in rabies diagnostic testing can create major complications, submission forms that are missing minor details are considered satisfactory. It also must be noted that the laboratory does not require evidence or proof of vaccination or other, similar information. Therefore, it is up to the veterinarians, the animal owner, complainant, or local health department to honestly and accurately fill out these fields to the best of their ability. Upon arrival to the rabies laboratory, all histories are labeled using an interagency identification code, specimens are necropsied, and slides are processed based on Centers for Disease Control and Prevention (CDC) and World Health Organization (WHO) recommendations [26]. Specimen histories are transcribed and verified into an in-house developed comprehensive laboratory information management system. We analyzed data extracted from the laboratory information management system with queries and other analytics in Microsoft Access 2019.

In this study, an Access search was performed to gather data on all cats and dogs from New York State submitted between January 1, 2008 and December 31, 2020. Specimens submitted directly to the New York City Laboratory were not included in this study. The query differentiated for the following: wild vs. owned animals, geographical location (latitude and longitude), vaccination history (current, not current, unvaccinated, unknown, or not answered), exposure status (bite, scratch, or contact with another human or domestic animal), direct fluorescent antibody (DFA) results (negative, positive, or indeterminate), and the antigenic viral variant of infection. The geographical information system (ArcGIS) was used to compile specimen demographics with New York State Civil boundary files, obtained through public records (ESRI, Redlands, California, USA).

### 2.2. Rabies Diagnostics and Variant Typing

Since 2008, the NYSDOH Rabies Laboratory has tracked and variant-typed all rabies- positive specimens for outbreak surveillance and host-spillover identification. In New York State, raccoon rabies variant (RRV) is the most common variant associated with rabies virus infection. Prior to March 2014, rabies variant testing was performed using a panel of monoclonal antibodies (MAb) [27].

In March 2014, the NYSDOH Rabies Laboratory transitioned from variant testing by MAbs to a more-sensitive method of molecular detection by RT-PCR. Diagnostic rabies testing was still performed with the gold standard, direct fluorescent antibody (DFA) [26], but follow-up raccoon variant typing was performed using the procedure outlined below.

Samples were prepared for extraction by suspending approximately 50 mg of macerated rabies-positive brain tissue in 1.0 mL of growth medium (GM). Total RNA was extracted using the Diagnostic Sample Preparation (DSP) Virus/Pathogen Kit (Qiagen, Hilden, Germany). Following the manufacturer’s protocol, a lysis buffer solution was prepared and aliquoted into screwcap (2.0 mL) tubes and 200 μL of brain homogenate subsample was added. Lysed samples were vortexed for approximately 15 s and placed in a heated bead bath (65 °C) for 15 min to complete viral inactivation and cellular digestion by proteinase K. Samples were then loaded onto the QIAsymphony SP platform (Qiagen, Hilden, Germany) for automated nucleic acid extraction and purification.

A master mix using the Quanta qScript Low-ROX RT-PCR kit (Quanta Biosciences, Gaithersburg, MD, USA) was prepared following the manufacturer’s instructions with raccoon rabies variant-specific primers. The assay oligonucleotides target a 63-base-pair (bp) region of the highly conserved nucleoprotein gene of raccoon rabies virus [28]. This previously published assay was slightly modified and optimized to adapt it to our in-house chemistry and RT-PCR platform. A total reaction volume of 20 μL (5 μL purified RNA; 15 μL of master mix) was templated onto 96-well plates for amplification and tested under standard cycling conditions using the Applied Biosystems 7500 FAST Real-Time PCR System (Thermo Fisher Scientific, Grand Island, NY, USA). Reactions were performed stepwise: 50 °C for 5 min, 95 °C for 30 s, and 45 cycles of 15 s at 95 °C and 1 min at 55 °C. Threshold and baseline values were manually set. Samples with cycle threshold values greater than 35 were repeated from extraction to ensure positivity. All samples were spiked with an exogenous transcript to check for inhibition, while a negative extraction control and no-template control (nuclease-free water) were run to ensure against contamination and non-specific amplification [29].

Specimens that did not amplify on this assay were sent for dideoxy sequencing at the Wadsworth Center sequencing core on an ABI 3130xl genetic analyzer (Thermo Fisher Scientific, Waltham, MA, USA). RT-PCR primers RABVD1 forward and RABVD2 reverse [29,30] were adapted for conventional PCR. They reliably amplify an approximate 700 base-pair section of the nucleoprotein gene that was compared to other rabies samples in the NCBI database using BLAST. The chemistry used was the Qiagen One-Step RT-PCR Kit (Qiagen, Hilden, Germany), following the manufacturer’s directions. PCR products were run on a 1% Tris-acetate-EDTA gel pre-stained with GreenGlo (Denville Scientific, Metuchen, NJ, USA) and visualized under ultraviolet illumination; bands were excised as appropriate and purified using Ambion spin columns (Thermo Fisher Scientific, Waltham, MA, USA).

### 2.3. Rabies Vaccines

The New York State Rabies Laboratory does not provide any vaccinations itself nor does it offer any veterinary advice to the public. These questions and concerns are referred to the New York State Bureau of Communicable Disease Control or to the county/local environmental public health office. A list of approved rabies vaccines is maintained by the National Association of Public Health Veterinarians [31], although our laboratory is not aware of what specific vaccine each submitted animal has received.

## 3. Results

### 3.1. Data and Demographics

#### 3.1.1. Feline Demographics

**Figure 1:**
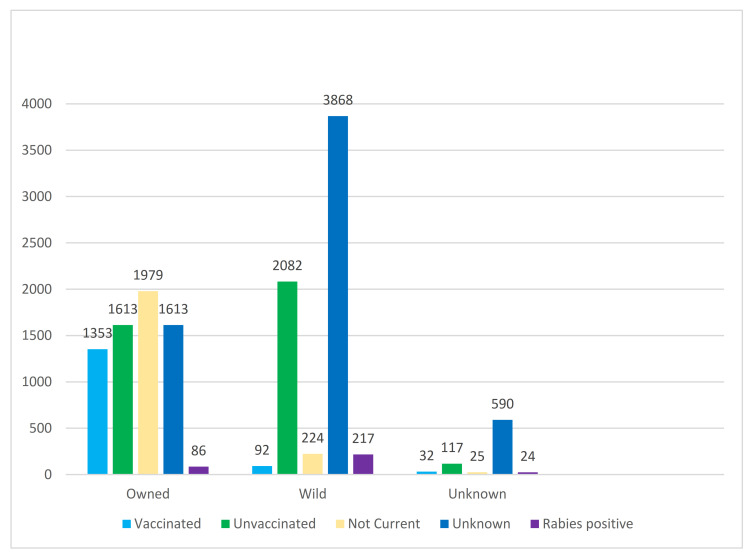
Ownership and vaccination status of cats submitted to the New York State Department of Health (NYSDOH) Rabies Laboratory.

In New York State, 13,915 cats were submitted (with a complete specimen history) for rabies testing between January 1, 2008 and December 31, 2020. Cats made up 17% of all specimens tested in the laboratory, and for comparison there were 76% more cats tested than dogs. A little over half the cats tested were reported as wild or unknown (7320) and a little under half as owned (6862). Of all cats submitted, only 1477 (10.6%) were reported to be current with rabies vaccination, including 124 with a wild or unknown ownership status. The majority (12,438) were reported as not current, unvaccinated, or having unknown vaccination status (Figure 1).

**Figure 2:**
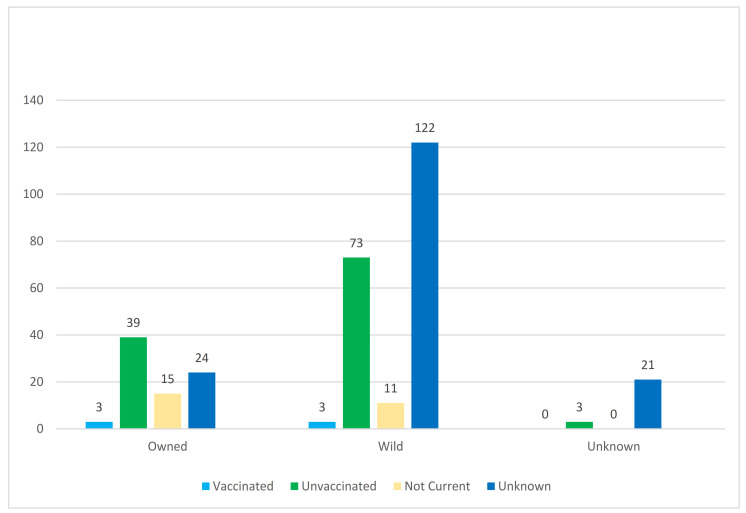
Ownership and vaccination status of rabies-positive cats submitted to the NYSDOH Rabies Laboratory.

Of the 13,915 cats with a vaccination status available, 314 (2.3%) tested positive for rabies, of which 273 (87%) had bitten or scratched a human and 87 (28%) had bitten another domestic animal. Of the positive cats, 206 (66%) were reported as wild and either had an unknown, unvaccinated, or not-current vaccination status (Figure 2).

Six rabid cats were reported as currently vaccinated. After further investigation, it was discovered that three of these cats were owned and three were wild. All the owned cats were recent animal-shelter adoptees and had received a rabies vaccine only days prior to being adopted. Of the wild cats, one was being maintained by a member of the community and died of rabies five days after vaccination while another belonged to a feral cat colony and had died of rabies two days after vaccination at a TNVR event; no supplemental data was available on the third positive wild cat.

**Figure 3:**
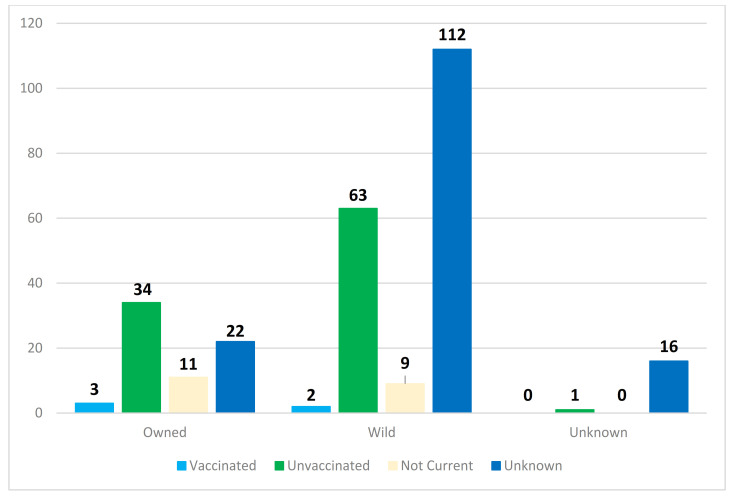
Ownership and vaccination status of rabies-positive cats submitted to the NYSDOH Rabies Laboratory that had either bitten or scratched a human.

In Figure 3, a breakdown of rabies-positive cats that specifically bit or scratched a human is shown. Notably, five of the six aforementioned rabid cats that were allegedly current on their rabies vaccinations did have contact with at least one human that required PEP. There were about 150% more wild rabid cats with human contact (186) compared to owned rabid cats with human contact (70).

**Figure 4:**
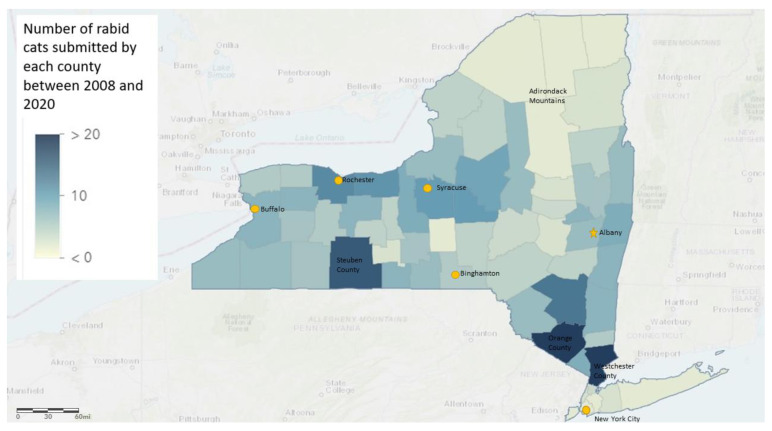
Number of rabies-positive cats based on New York county data (excluding New York City and Long Island).

A geographical representation of where the rabid cats resided can be seen in Figure 4. Westchester, Orange, and Steuben counties submitted the most rabid cats. The sparsely populated counties in the Adirondack Mountains of northern New York generally had the fewest rabid cat specimens.

#### 3.1.2. Canine Demographics

**Figure 5:**
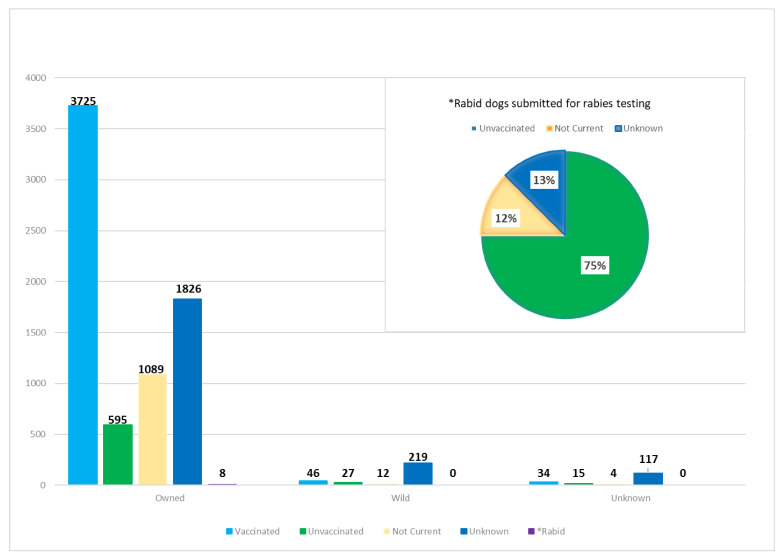
Ownership and vaccination status of dogs submitted to the NYSDOH Rabies Laboratory. Inset shows details of the eight dogs that were positive over the same 2008–2020 timeframe.

Considerably fewer dogs (with a complete specimen history) were submitted during the same time period, totaling 7717 (10%) of all specimen samples. More than 94% (7243) of the dogs were reported as owned, which is substantially more than in the cat submissions. Of the submitted total, 304 (4%) were reported as wild and 3805 of all dogs submitted for testing were currently vaccinated (Figure 5). In fact, dogs were 61% more likely than cats to be vaccinated regardless of their ownership status. Between 2008–2020, eight dogs tested positive for rabies at the New York State Wadsworth Center Rabies Laboratory. All eight were reported as owned; six were unvaccinated, one was allegedly not current, and one had an unknown vaccination history. All eight had bitten or scratched a human. Four of the eight reportedly exposed another domestic animal, and the other half had an unknown history of domestic animal exposure.

### 3.2. Variant Typing

#### 3.2.1. Variant Results in Felines

Rabies variant results were available on 270 (86%) of the cats submitted for testing. Of the 270 cats, 269 were infected with raccoon rabies variant and one was infected with north central skunk variant. The sample with north central skunk variant was a cat submitted to the laboratory in December 2016 after it had become neurologic, attacked a dog, and bit/scratched three people. This cat was negative by RT-PCR for raccoon variant, so an approximate 700 base-pair section of the nucleoprotein gene was dideoxy sequenced to characterize the virus and determined to be consistent with north central skunk variants found in Genbank. An epidemiological investigation revealed that the kitten was originally 1 of 10 from a litter born on a farm in Rose Creek, Minnesota. The owner lived in Ames, Iowa and had adopted the animal. It had been transported to a family member’s home in Johnsonville, NY that the owner visited for a winter/holiday vacation.

#### 3.2.2. Variant Results in Canines

Of the eight rabies-positive dogs, seven were infected with raccoon rabies variant and one was infected with arctic fox variant. The dog with arctic fox variant was a 6-month old puppy that originally resided in Kangirsuk, Quebec, Canada. It had been transported back to the United States without a health certificate or rabies vaccination history. As with the translocated cat, an approximate 700 base-pair section of the nucleoprotein gene was dideoxy-sequenced and found to be consistent with the arctic fox variant. Due to the epidemiological interest in this case, whole genome sequencing (WGS) was performed and uploaded to GenBank with accession number MN418166, as described here [32]. Phylogenetic analysis nested this specific virus in fox lineage III as expected based on its geographical location near the Arctic Circle [33].

## 4. Discussion

### 4.1. Overview

Despite access to highly efficacious rabies vaccines for domestic animals, every year hundreds of domestic animals are diagnosed with rabies in the United States. Nationwide between 2008 and 2017, 2503 cats and 714 dogs tested positive for rabies virus infection. In New York State, 314 cats and 8 dogs submitted between 2008 and 2020 were positive for rabies. For comparison, the NYSDOH Rabies Laboratory receives approximately 200 positive terrestrial vector species with raccoon variant and 100 positive *Chiroptera* with bat variants every year.

### 4.2. Animal Health Law

New York State public health law requires that all dogs, cats, and ferrets be current on rabies vaccination [34]. However, rabies vaccine requirements vary widely among states. For instance, Ohio and Kansas, among others, have no rabies vaccine requirements for cats [9,35]. In support of New York State’s mandatory vaccination requirement, county health departments provide several free rabies vaccine clinics throughout the year, most often quarterly. Despite the availability of no-cost rabies vaccination, thousands of owned animals remain unvaccinated or under-vaccinated against rabies.

Regardless of the laws in place, how they are applied in practice depends greatly on how pet care-takers regard their animals. Dogs are more commonly thought of as members of the family (94% owned, 49% vaccinated), while cats are not (48% owned, 11% vaccinated). Occasionally, groups of people or neighborhoods claim unofficial possession of feral animal populations, often referred to as community ownership. In these situations, animals may be provided food but not necessarily veterinary care such as rabies vaccinations. Feral cat colonies are not necessarily common in New York State but do exist. In other parts of the world, community ownership of dogs can be widespread and often coincides with regions where canine rabies is endemic [34,35,36].

### 4.3. The Under-Vaccinating Dilemma

Cost, accessibility, educational level of care takers, personal beliefs, and medical concerns compound the lack of proper rabies vaccinations. In Africa and Asia, where canine rabies is endemic, cost and accessibility are the major factors limiting vaccination, but these are much less of a problem in the United States [37,38]. Cat owners specifically underestimate their pets’ risk for rabies despite their vulnerability to terrestrial rabies variants, especially in urban centers. In 2017, of the 293 specimens submitted from the five boroughs of New York City, 78 were cats suspected of having rabies, second only to 104 raccoons. Of those 78 cats, 2 were positive and infected with RRV [39]. Additionally, 133 raccoons were infected with rabies following an outbreak in Central Park in 2009, demonstrating the transmission potential in major metropolitan centers [40]. The public must be reminded that rabies exists in this urban setting and transmission to unvaccinated or under-vaccinated pets is possible.

### 4.4. Feline Rabies

The number of cat rabies cases are often greatest in states where RRV is endemic [18]. This is further supported by the overwhelming proportion of rabid cats in New York State that are infected with raccoon rabies as opposed to a bat rabies variant. Interestingly in 2020, our laboratory received 10 raccoons that had known incidents with cats, meanwhile, we received 536 bats that had contact with cats. With that large of an imbalance, the absence of cats infected with bat rabies virus variants is a noteworthy However, we recognize that this is skewed to an unknown extent because the actual number of raccoon–cat interactions would be much higher as they occur outside the home, often when humans are not near.

Undeniably, households with indoor-outdoor cats that are unvaccinated or not currently vaccinated are at an increased risk of rabies exposure. Additionally, households with small children may not be aware of a rabies exposure, as children do not always disclose incidents of bites or scratches [25,41,42]. The first and only confirmed case of human rabies attributed to a cat occurred in Minnesota in 1975 [43]. In 2011, an 8-year-old child was presumptively diagnosed with rabies; an epidemiological investigation revealed she had contact with multiple free-roaming unvaccinated cats, suggesting, but not confirming, cats as a possible rabies vector source [25]. Although human rabies cases in the US are generally attributed to bat rabies, cat exposures make up a large percentage of individuals administered PEP [44,45]. A 2010 study reported that cat exposures accounted for more administrations of PEP than exposures to any other species, including wildlife [46]. In the US, 16% percent of the 55,000 individuals who receive PEP annually do so following contact with a cat, equating to 8800 individuals with an average total cost of $33,440,000 [47].

We found that 62% of rabid cats in New York State were unowned and had no proof of vaccination, and canbe described as free-roaming cats. While law makers and animal advocacy groups can agree that mass euthanasia of these animals is not pragmatic, their control remains a conundrum for public health officials. Despite implementing trap-neuter-vaccinate-return (TNVR) programs that include rabies vaccination, it is estimated that less than 10% of feral or free-roaming cats are ever trapped. Individuals that feed colonies are at an increased exposure risk, since feeding generally increases colony size and attracts wildlife, including rabies reservoir species [22]. Additionally, most cats are only trapped once and unlikely to receive additional rabies vaccine, thereby diminishing immunity against rabies over a lifetime. TNVR programs have increased in popularity but their outcomes are uncertain. Due to the addition of unsterilized cats into a colony, varying degrees of TNVR maintenance, and variable implementation rates, TNVR may not effectively control feral cat populations [22]. However, two subsequent studies demonstrated a decrease of free-roaming cat intake and euthanasia in designated study sites [48,49]. The data regarding the long-term success of TNVR in community cat populations are uncertain. Rather, a multifactorial approach including responsible pet ownership, universal rabies vaccination of pets, and reductions in the number of strays will remain a keystone component of measures to control rabies exposure risk [22].

### 4.5. Canine Rabies

Since the eradication of dog rabies in the United States, dogs play a limited rolein the perpetuation of rabies in domestic animals. While canine variant rabies itself has not been detected in the United States for decades and was widely considered to be eradicated in 2007 [50], dogs almost always contract the local terrestrial variant. These positive infections often lead to preventable euthanasia of unvaccinated animal contacts, long quarantines, and human PEP [51,52]. Globally, however, canine rabies remains dangerously endemic and is directly correlated with poverty, lack of education, and poor access to healthcare [53].

### 4.6. Final Thoughts

In the United States, the scarcity of rabies transmission from domestic animals to humans is often used to discount the overall risk of rabies. Howeverit is not a justifiable defense fordisregarding responsible pet ownership. Animals play integral roles in the lives of millions and several research studies have identified a positive correlation between emotional well-being and pet ownership [54,55,56]. Efforts to preserve this effective relationship are supported by public health initiatives like One Health, which necessitate partnerships between veterinarians, animal advocates, and other public health representatives to better manage guidelines encouraging responsible pet ownership through reductions in the number of unwanted and abandoned cats, increasing programs available to encourage ethical adoption, and improving rabies vaccination rates in all domestic animals.

## Data Availability

Data is contained within the article.
